# Supplementary material for: Convergence of plasmid-driven virulence and antibiotic resistance in Escherichia coli
Source: Nat Commun. 2025 Dec 10;17:505. doi: 10.1038/s41467-025-67202-9 (PMC12804835; doi:10.1038/s41467-025-67202-9)
Supplement: Supplementary file 1 — Supplementary Information [file 41467_2025_67202_MOESM1_ESM.pdf]

Supplementary Figures

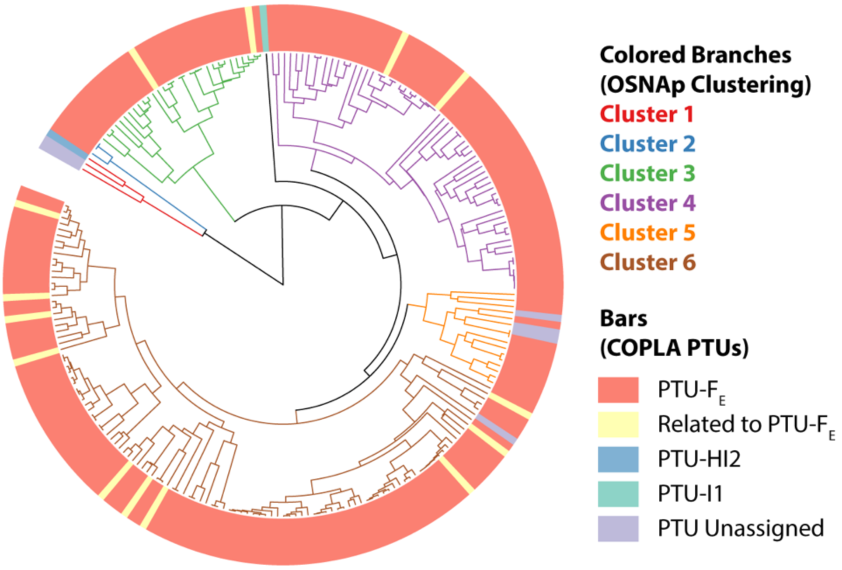

Supplementary Figure 1. Plasmid taxonomic unit (PTU) assignments of 233 ColVLPs generated using COPLA.

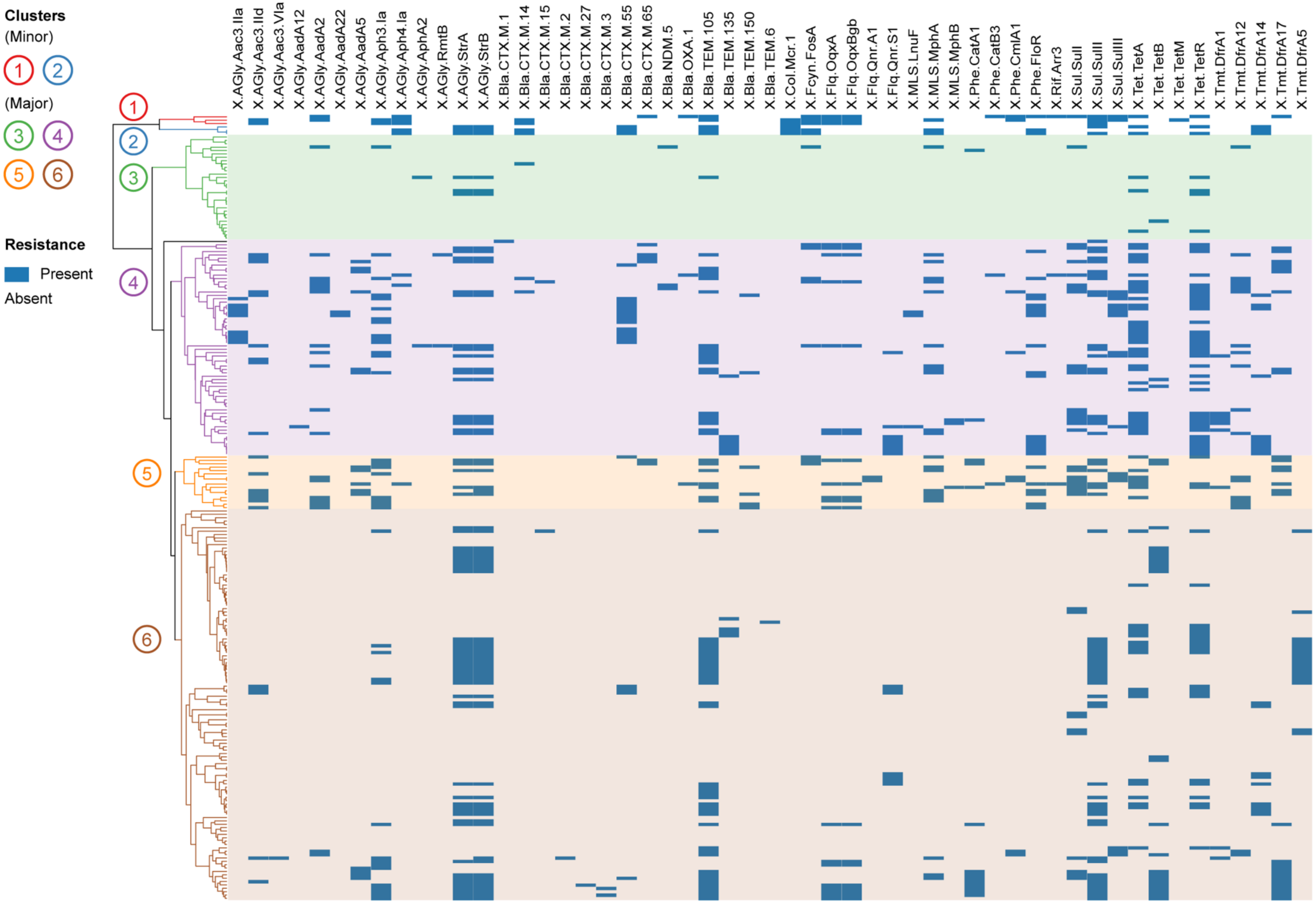

Clusters
(Minor)
1
2
(Major)
3
4
5
6
Resistance
Present
Absent
X.AGly.Aac3.IIa
X.AGly.Aac3.IId
X.AGly.Aac3.VIa
X.AGly.AadA12
X.AGly.AadA2
X.AGly.AadA22
X.AGly.AadA5
X.AGly.Aph3.Ia
X.AGly.Aph4.Ia
X.AGly.AphA2
X.AGly.RmtB
X.AGly.StrA
X.AGly.StrB
X.Bla.CTX.M.1
X.Bla.CTX.M.14
X.Bla.CTX.M.15
X.Bla.CTX.M.2
X.Bla.CTX.M.27
X.Bla.CTX.M.3
X.Bla.CTX.M.55
X.Bla.CTX.M.65
X.Bla.NDM.5
X.Bla.OXA.1
X.Bla.TEM.105
X.Bla.TEM.135
X.Bla.TEM.150
X.Bla.TEM.6
X.Col.Mcr.1
X.Fcyn.FosA
X.Flq.OqxA
X.Flq.OqxBgb
X.Flq.Qnr.A1
X.Flq.Qnr.S1
X.MLS.LnuF
X.MLS.MphA
X.MLS.MphB
X.Phe.CatA1
X.Phe.CatB3
X.Phe.CmlA1
X.Phe.FloR
X.Rif.Arr3
X.Sul.SulI
X.Sul.SulII
X.Sul.SulIII
X.Tet.TetA
X.Tet.TetB
X.Tet.TetM
X.Tet.TetR
X.Tmt.DfrA1
X.Tmt.DfrA12
X.Tmt.DfrA14
X.Tmt.DfrA17
X.Tmt.DfrA5

Supplementary Figure 2. AMR genes within the 233 ColVLPs. AMR genes were identified using ABRicate v0.8 against the ARG-ANNOT database (1) using a 100% query length threshold and plotted against the 233 ColVLP cladogram as described in Figure 1.

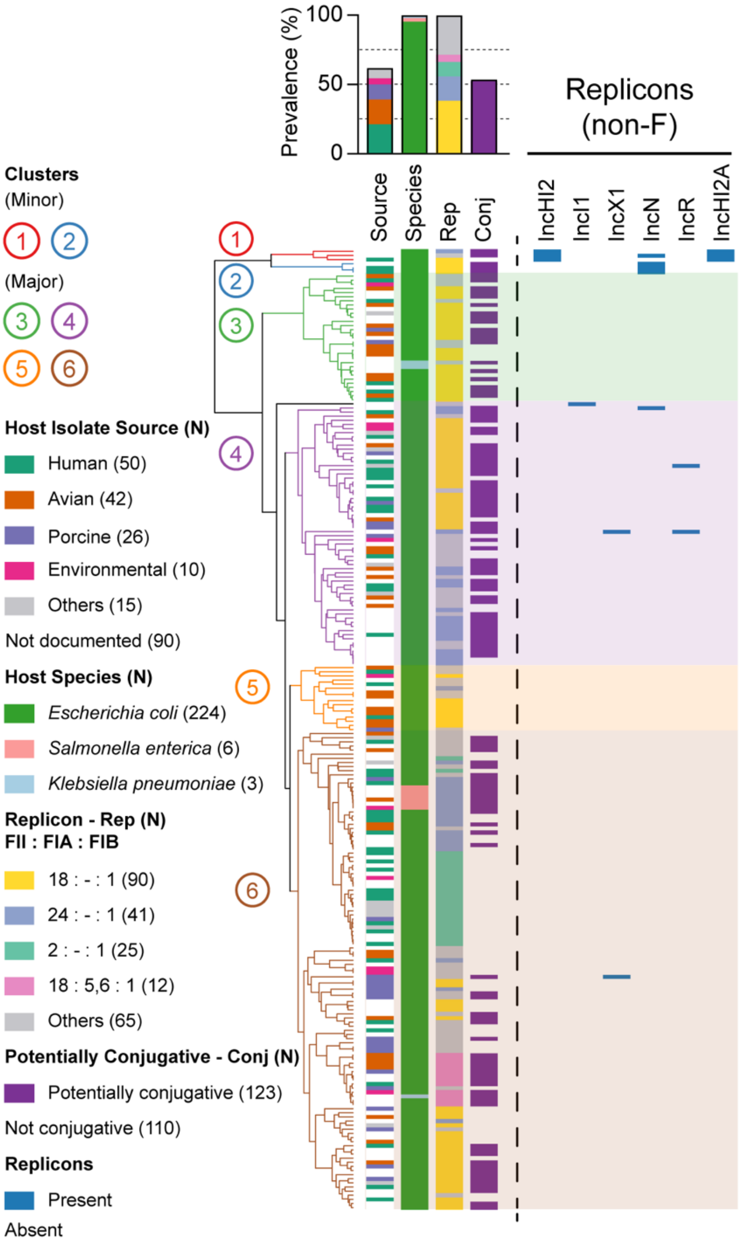

Supplementary Figure 3. Non-IncF replicons within the 233 ColVLPs. Additional replicons were identified using the PlasmidFinder database as a BLASTn query at a 90% identity and alignment length threshold and plotted against the 233 ColVLP cladogram as described in Figure 1.

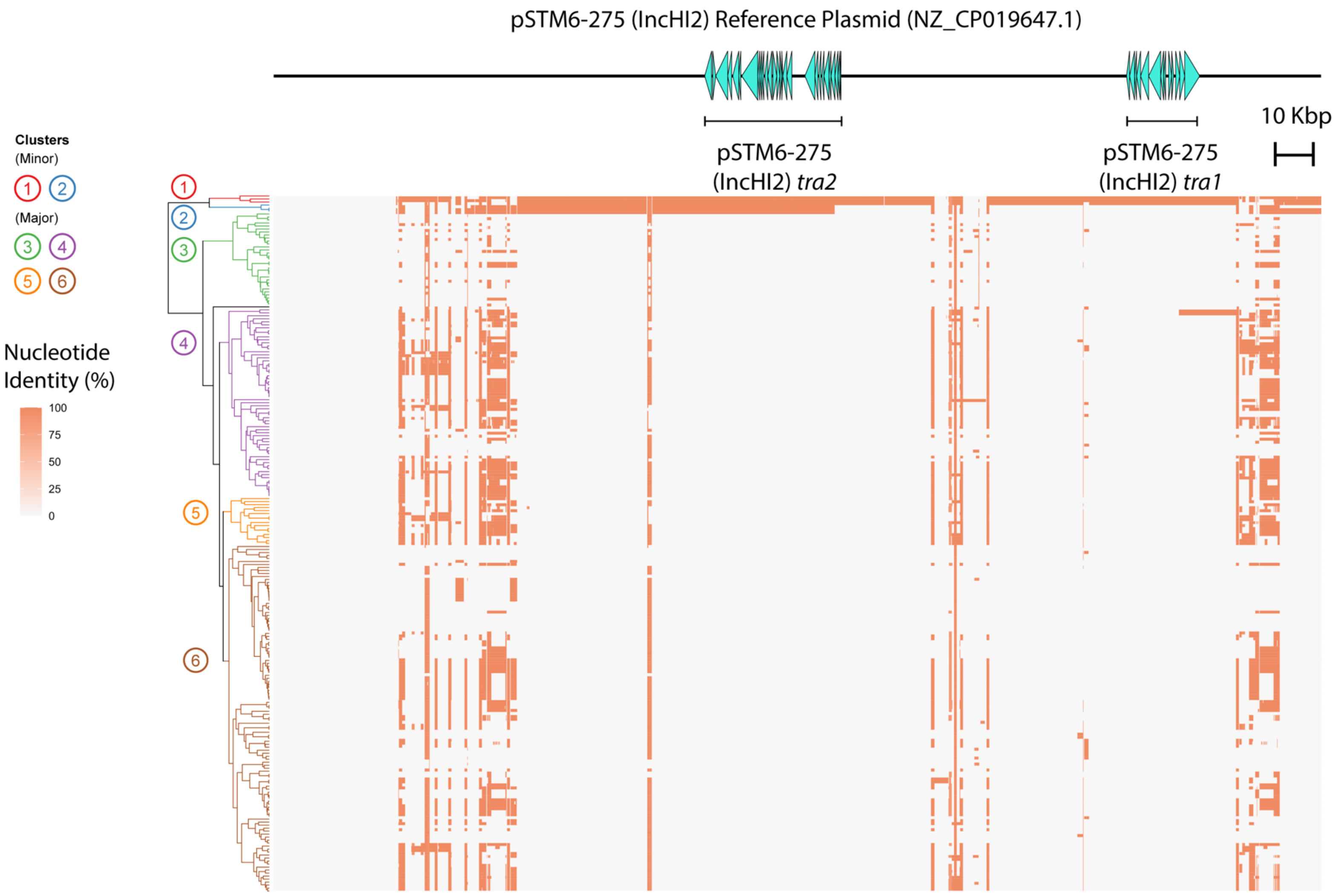
pSTM6-275 (IncHI2) Reference Plasmid (NZ_CP019647.1)
10 Kbp
pSTM6-275 (IncHI2) tra2
pSTM6-275 (IncHI2) tra1
Clusters
(Minor)
1 2
(Major)
3 4
5 6
Nucleotide Identity (%)
100
75
50
25
0

Supplementary Figure 4. Nucleotide identity (%) heatmap of 150-bp bins from the IncHI2 reference plasmid pSTM6-275 against the 233 ColVLP cladogram. Bins were used in a BLASTn query against the 233 ColVLPs at a 75% identity and length threshold.

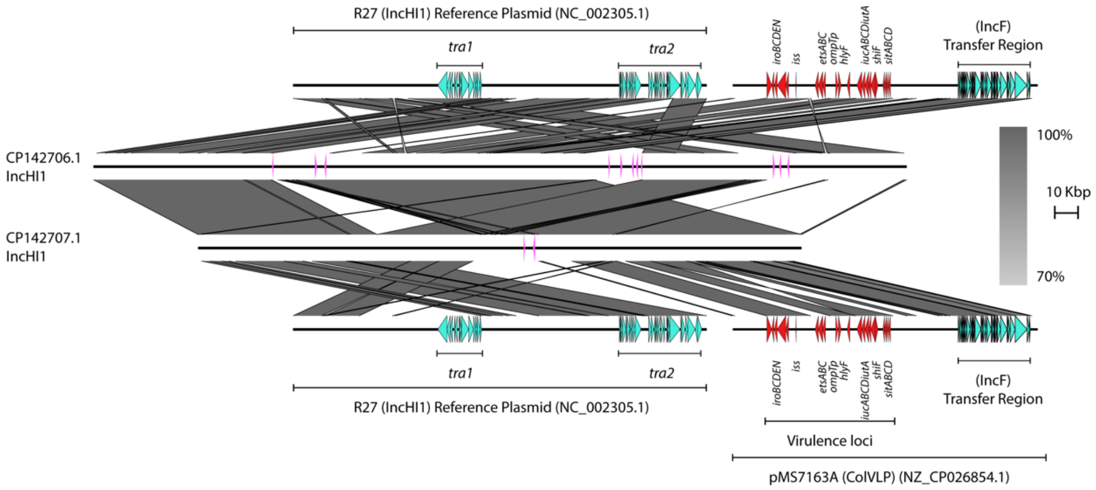

Supplementary Figure 5. Pairwise comparison of the IncHI1/ColVLP hybrids. Features are coloured as follows: ColVLP-associated virulence loci – red; Transfer genes – blue; IS26 elements – pink.

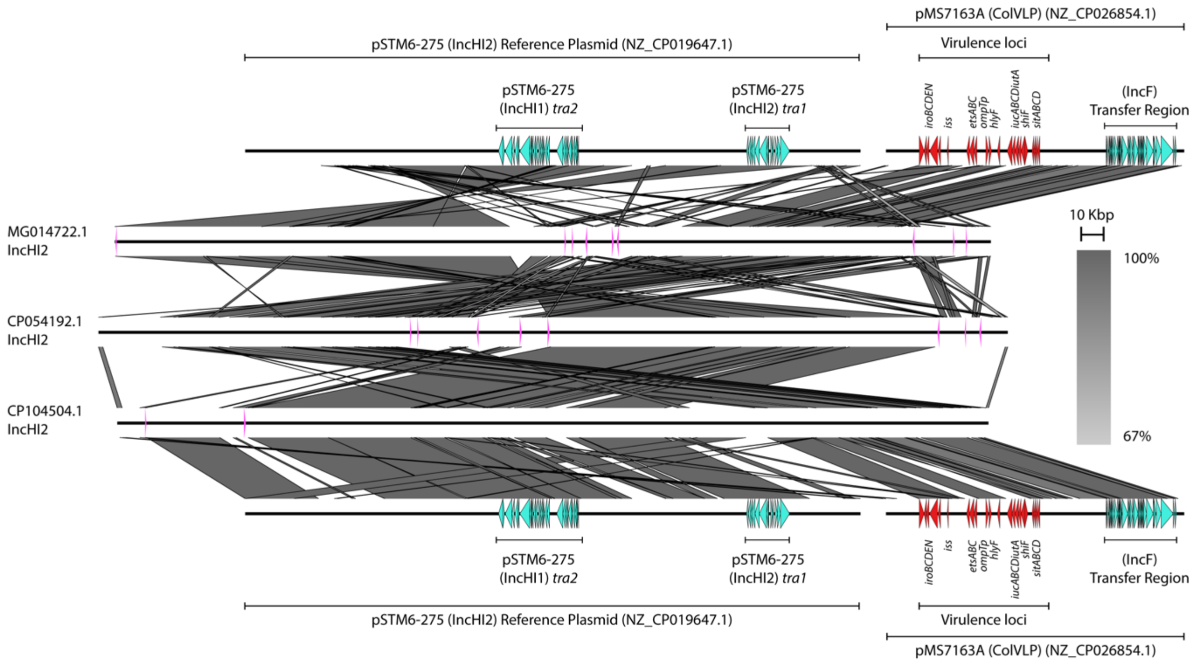

Supplementary Figure 6. Pairwise comparison of the IncHI2/ColVLP hybrids. Features are coloured as follows: ColVLP-associated virulence loci – red; Transfer genes – blue; IS26 elements – pink.

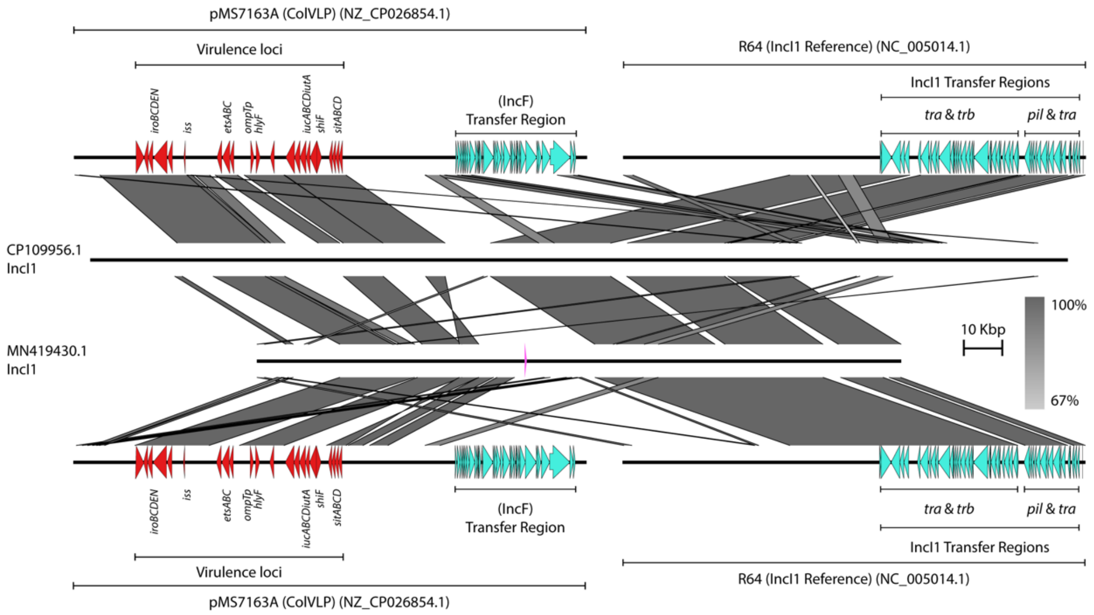

Supplementary Figure 7. Pairwise comparison of the IncI1/ColVLP hybrids. Features are coloured as follows: ColVLP-associated virulence loci – red; Transfer genes – blue; IS26 elements – pink.

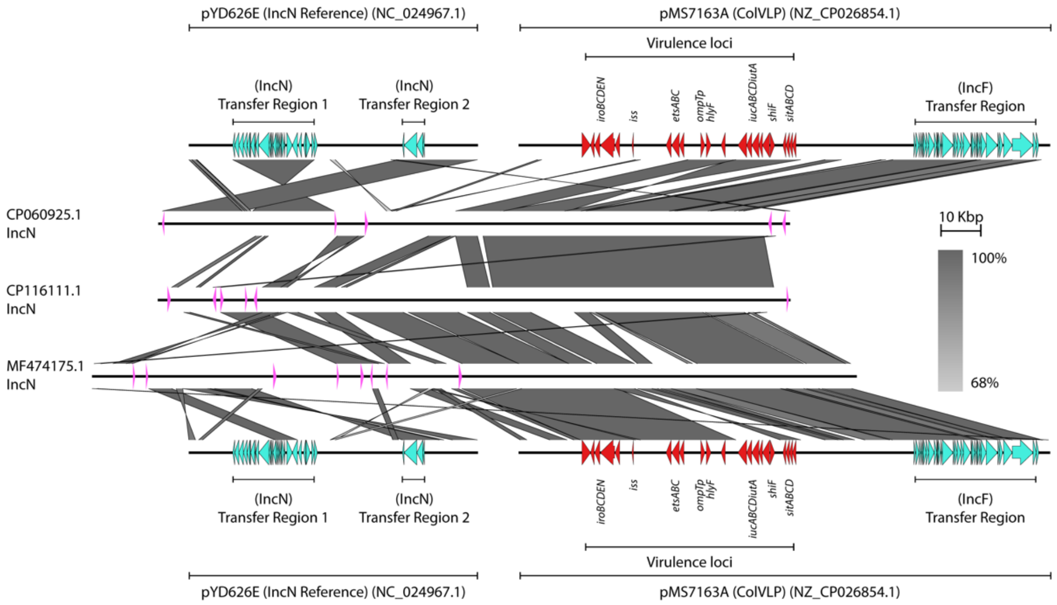

Supplementary Figure 8. Pairwise comparison of the IncN/ColVLP hybrids. Features are coloured as follows: ColVLP-associated virulence loci – red; Transfer genes – blue; IS26 elements – pink.

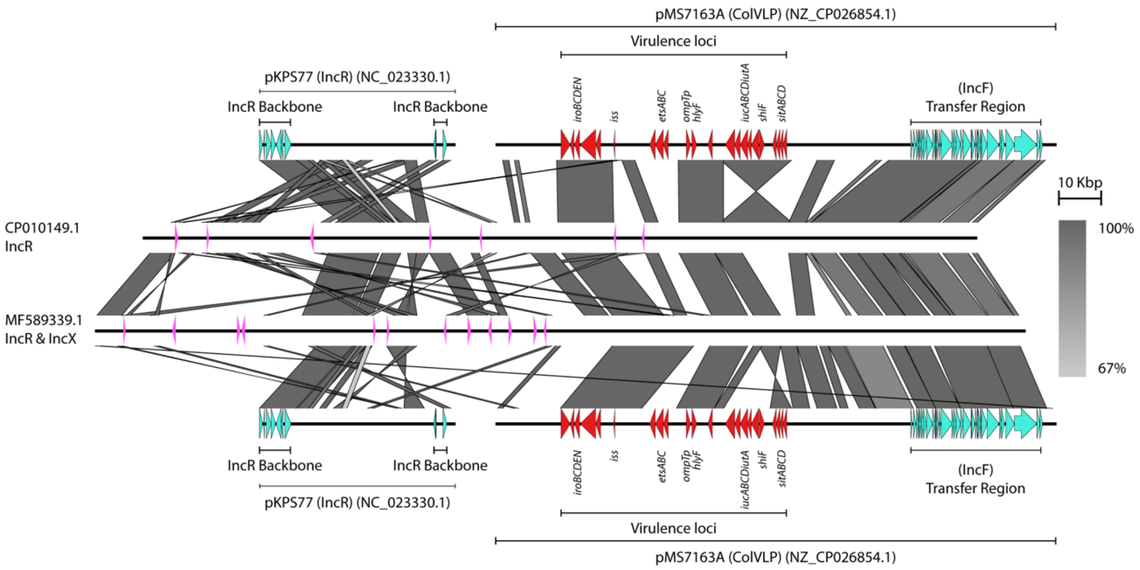

Supplementary Figure 9. Pairwise comparison of the IncR/ColVLP hybrids. Features are coloured as follows: ColVLP-associated virulence loci – red; Transfer genes – blue; IS26 elements – pink.

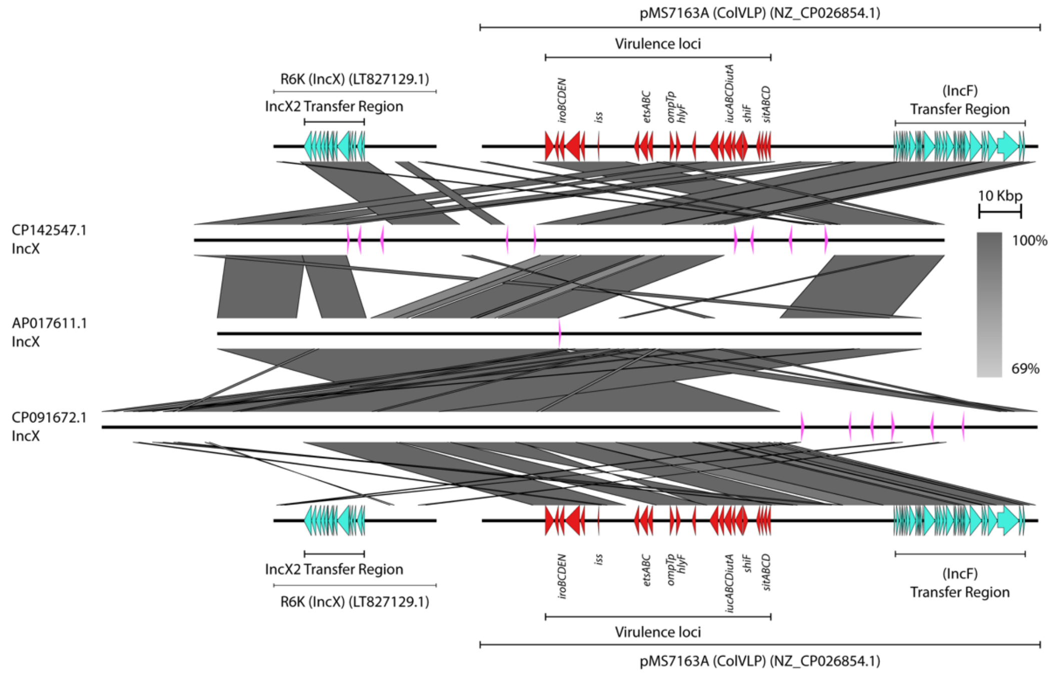

Supplementary Figure 10. Pairwise comparison of the IncX/ColVLP hybrids. Features are coloured as follows: ColVLP-associated virulence loci – red; Transfer genes – blue; IS26 elements – pink.

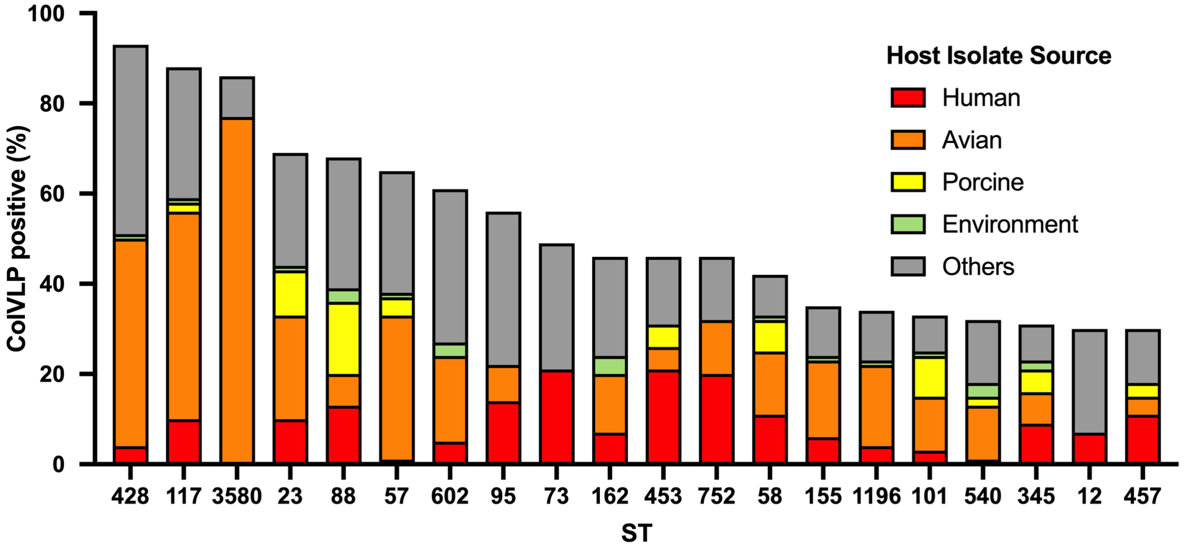

Supplementary Figure 11. ColVLP carriage in ExPEC based on Sequence Type (ST). Shown are the top 20 STs containing ColVLPs in the 100ST dataset (comprising 10,000

genomes made up of 100 genomes from each of the top 100 STs in Enterobase). Colours indicate the host isolation source, with the most common defined sources being human and animal (primarily poultry) hosts. ColVLPs were determined using the criteria proposed by Liu C. M. et al. (2).

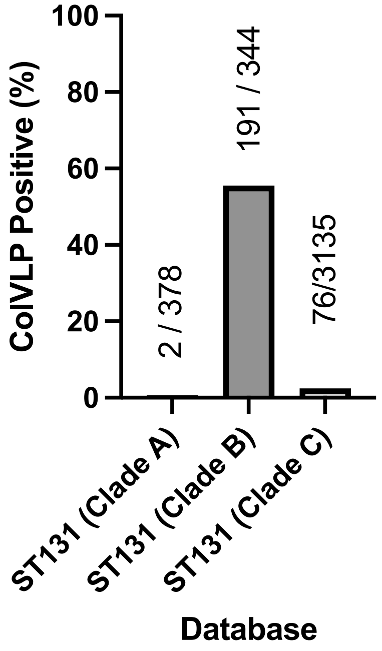

Supplementary Figure 12. ColVLP carriage in ST131. ST131 draft genomes (n = 3,857) were queried for ColVLP carriage using the criteria proposed by Liu C. M. et al. (2).

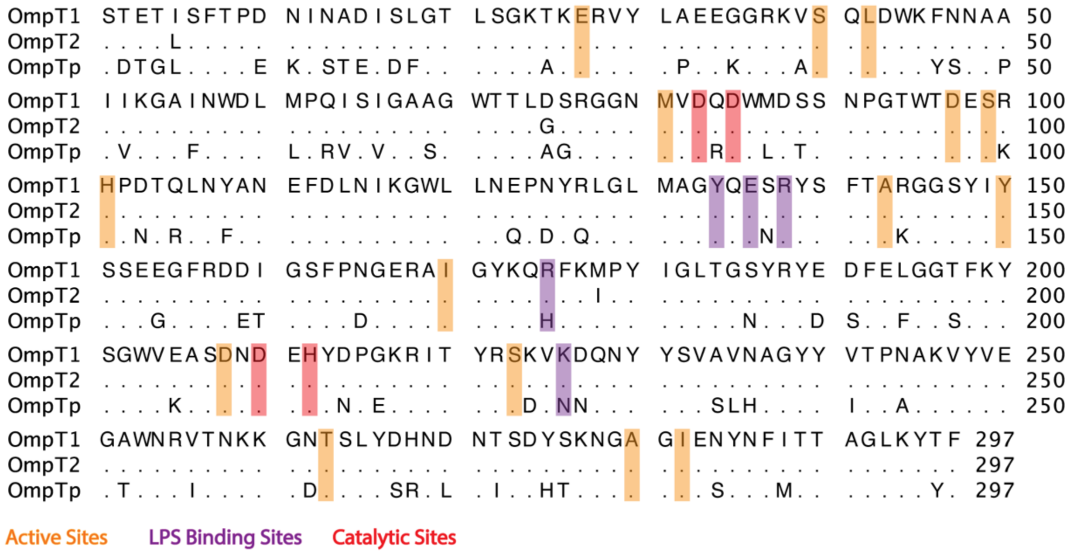

Supplementary Figure 13. Amino acid sequence alignment of OmpT1, OmpT2, and OmpTp. Alignments of OmpT1, OmpT2, and OmpTp sequences without the signal peptide were performed using CLC Main Workbench. Key residues are highlighted as follows: Active sites – yellow; Catalytic sites – red; LPS binding sites – purple.

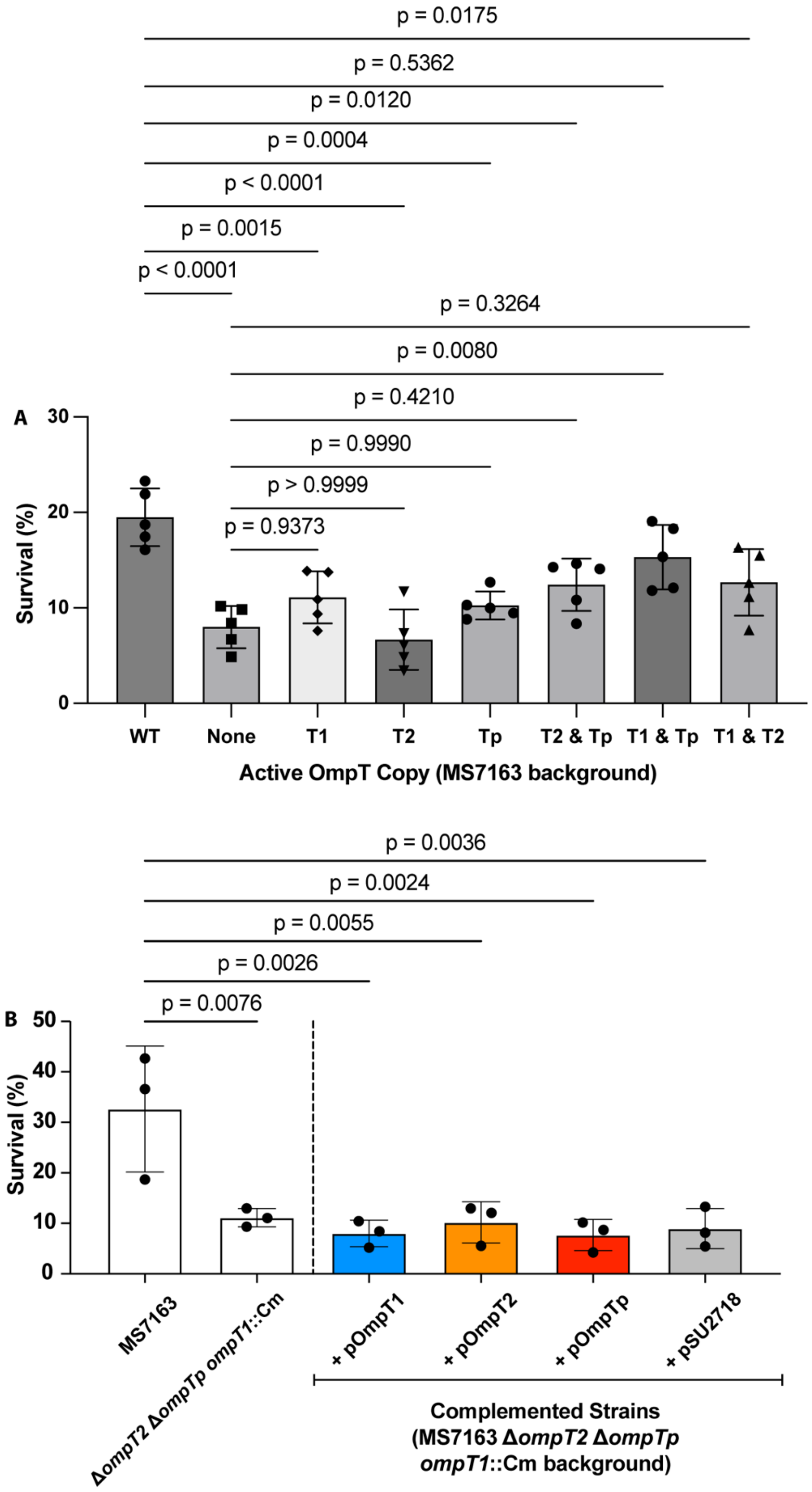

Supplementary Figure 14. (A) Survival of MS7163 derivatives against 0.05mg/mL LL-37. Wildtype MS7163 expresses three OmpT variants: OmpT1, OmpT2, OmpTp. Data is

represented as Mean ± SD of five biological replicates. **(B) Survival of MS7163 Δ*ompT2* Δ*ompTp ompT1*::Cm + pOmpT complement derivatives against 0.05mg/mL LL-37.** Data is represented as Mean ± SD of three biological replicates. Significance was determined using a One-way ANOVA with Dunnett's multiple comparisons. Survival of strains after LL-37 incubation is expressed as a percentage of CFU count in PBS control at time 0.

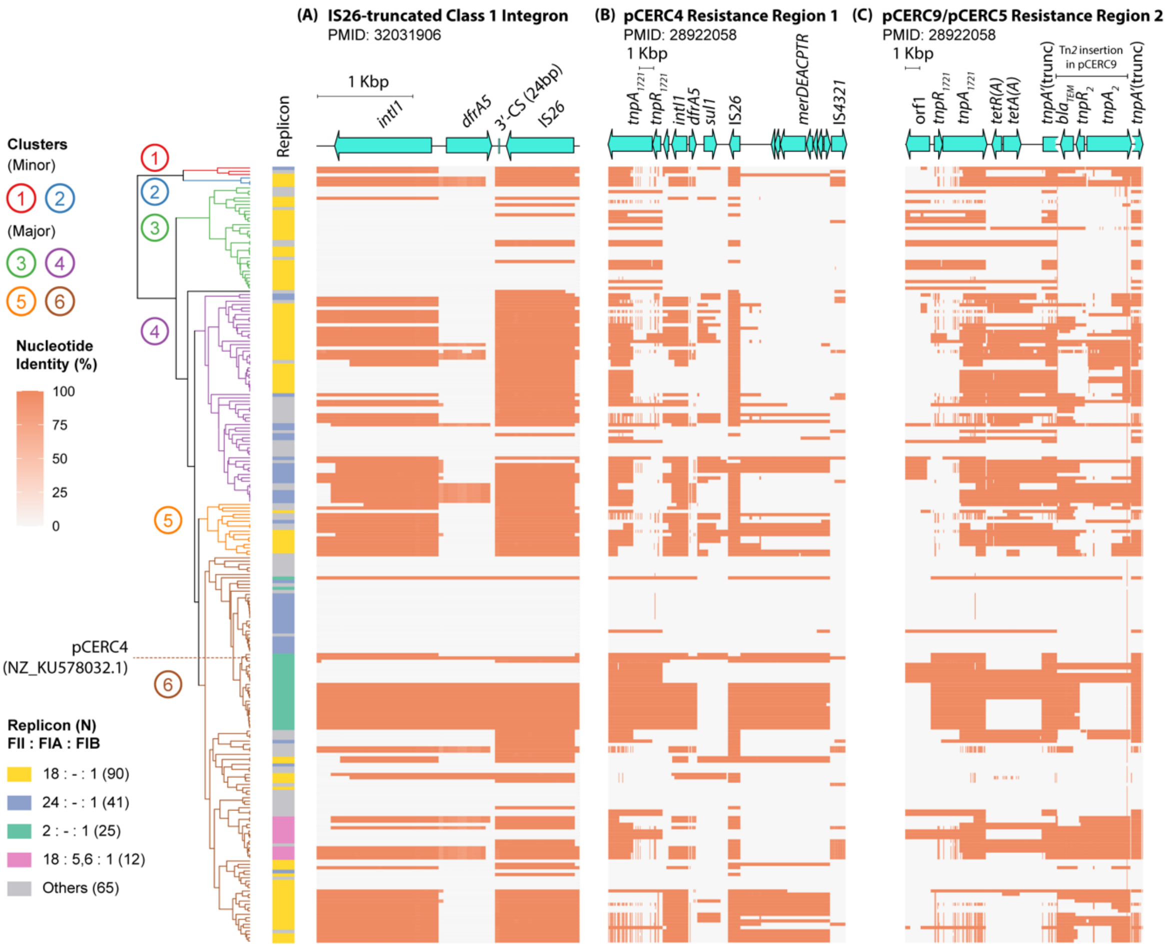

Supplementary Figure 15. Nucleotide identity (%) heatmap of (A) Class 1 integron and the IS*26* truncation; (B) Plasmid pCERC4 Resistance Region 1; (C) Plasmid pCERC9/pCERC5 Resistance Region 2 against the 233 ColVLP cladogram. Heatmaps were generated using 50-bp bins from reference molecular signatures as a BLASTn query against the 233 ColVLPs, at 75% percent identity and alignment length thresholds. The IS*26*-truncated class 1 integron molecular signature was identified by McKinnon J. et al. (3), while

pCERC4/pCERC9/pCERC5 resistance regions were identified by Moran R. A. and R. M. Hall (4). The FII-2/FIB-1 plasmid pCERC4 is highlighted as reference.

References

1. Gupta SK, Padmanabhan BR, Diene SM, Lopez-Rojas R, Kempf M, Landraud L, Rolain JM. 2014. ARG-ANNOT, a new bioinformatic tool to discover antibiotic resistance genes in bacterial genomes. Antimicrob Agents Chemother 58:212-20.
2. Liu CM, Stegger M, Aziz M, Johnson TJ, Waits K, Nordstrom L, Gauld L, Weaver B, Rolland D, Statham S, Horwinski J, Sariya S, Davis GS, Sokurenko E, Keim P, Johnson JR, Price LB. 2018. *Escherichia coli* ST131-*H*22 as a Foodborne Uropathogen. mBio 9:e00470-18.
3. McKinnon J, Roy Chowdhury P, Djordjevic SP. 2020. Molecular Analysis of an IncF ColV-Like Plasmid Lineage That Carries a Complex Resistance Locus with a Trackable Genetic Signature. Microbial Drug Resistance 26:787-793.
4. Moran RA, Hall RM. 2017. Evolution of Regions Containing Antibiotic Resistance Genes in FII-2-FIB-1 ColV-Colla Virulence Plasmids. Microbial Drug Resistance 24:411-421.
