# Supplementary material for: Convergence of plasmid-driven virulence and antibiotic resistance in Escherichia coli
Source: Nat Commun. 2025 Dec 10;17:505. doi: 10.1038/s41467-025-67202-9 (PMC12804835; doi:10.1038/s41467-025-67202-9)
Supplement: Supplementary file 2 — Description of Additional Supplementary Files [file 41467_2025_67202_MOESM2_ESM.pdf]

Description of additional Supplementary Data files

Supplementary Data 1. Features associated with the 20 ColVLP hybrids

Supplementary Data 2. Strains and plasmids

Supplementary Data 3. Primers used

Supplementary Data 4. Features of 233 ColVLPs

Supplementary Data 5. Metadata of the 100ST Database

Supplementary Data 6. Homologs of *ompT* in the 1377 complete *E. coli* genomes

Supplementary Data 7. Additional replicons found in the ST95 draft genomes database

Supplementary Data 8. Additional replicons found in the ST131 Clade B draft genomes database
