# Supplementary material for: Convergence of plasmid-driven virulence and antibiotic resistance in Escherichia coli
Source: Nat Commun. 2025 Dec 10;17:505. doi: 10.1038/s41467-025-67202-9 (PMC12804835; doi:10.1038/s41467-025-67202-9)
Supplement: Supplementary file 4 — Reporting Summary [file 41467_2025_67202_MOESM4_ESM.pdf]

nature portfolio

Corresponding author(s): Mark Schembri, Minh-Duy Phan

Last updated by author(s): Oct 27, 2025

Reporting Summary

Nature Portfolio wishes to improve the reproducibility of the work that we publish. This form provides structure for consistency and transparency in reporting. For further information on Nature Portfolio policies, see our Editorial Policies and the Editorial Policy Checklist.

Statistics

For all statistical analyses, confirm that the following items are present in the figure legend, table legend, main text, or Methods section.

n/a	Confirmed	
☐	☒	The exact sample size (*n*) for each experimental group/condition, given as a discrete number and unit of measurement
☐	☒	A statement on whether measurements were taken from distinct samples or whether the same sample was measured repeatedly
☐	☒	The statistical test(s) used AND whether they are one- or two-sided<br>*Only common tests should be described solely by name; describe more complex techniques in the Methods section.*
☒	☐	A description of all covariates tested
☐	☒	A description of any assumptions or corrections, such as tests of normality and adjustment for multiple comparisons
☐	☒	A full description of the statistical parameters including central tendency (e.g. means) or other basic estimates (e.g. regression coefficient) AND variation (e.g. standard deviation) or associated estimates of uncertainty (e.g. confidence intervals)
☐	☒	For null hypothesis testing, the test statistic (e.g. *F*, *t*, *r*) with confidence intervals, effect sizes, degrees of freedom and *P* value noted<br>*Give P values as exact values whenever suitable.*
☒	☐	For Bayesian analysis, information on the choice of priors and Markov chain Monte Carlo settings
☐	☒	For hierarchical and complex designs, identification of the appropriate level for tests and full reporting of outcomes
☒	☐	Estimates of effect sizes (e.g. Cohen's *d*, Pearson's *r*), indicating how they were calculated

Our web collection on statistics for biologists contains articles on many of the points above.

Software and code

Policy information about availability of computer code

Data collection	No software was used for data collection
Data analysis	Data analyses were performed using the following software:<br><br>R version 4.3.2 (2023-11-01), available at https://cran.r-project.org/bin/macosx/<br>The R Stats Package 4.3.2 (2023-11-01), available at https://cran.r-project.org/bin/macosx/<br>ORF-based binarized structure network analyses tool, available at DOI:10.1016/j.plasmid.2019.102477<br>SplitsTree v6.3.10, available at https://github.com/husonlab/splitstree6<br>COPLA, available at https://github.com/santirdnd/COPLA<br>Prism10 (Version 10.2.0), available at https://www.graphpad.com/scientific-software/prism/<br>BLASTn (Version 2.14.0+), available at https://www.ncbi.nlm.nih.gov/guide/howto/run-blast-local/<br>Clustal Omega (Version 1.2.3), available at http://www.clustal.org/omega/<br>ABRicate version 0.8.10, available at https://github.com/tseemann/abricate<br>MLST version 2.23.0, available at https://github.com/tseemann/mlst<br>Easyfig version 2.2.2, available at https://mjsull.github.io/Easyfig/<br>FastTree version 2.1, available at https://morgannprice.github.io/fasttree/<br>ChimeraX version 1.6.1, available at https://www.cgl.ucsf.edu/chimerax/<br>ColabFold version 1.5.2, available at https://colab.research.google.com/github/sokrypton/ColabFold/blob/main/AlphaFold2.ipynb<br>Analyst TF version 1.6, available at https://sciex.com/products/software/analyst-tf-software

Cytoscape version 3.9.1, available at https://cytoscape.org/
CLC Main Workbench version 23.0.4, available at https://digitalinsights.qiagen.com/products-overview/discovery-insights-portfolio/analysis-and-visualization/qiagen-clc-main-workbench/
MultiQuant version 3.0.3, available at https://sciex.com/products/software/multiquant-software

For manuscripts utilizing custom algorithms or software that are central to the research but not yet described in published literature, software must be made available to editors and reviewers. We strongly encourage code deposition in a community repository (e.g. GitHub). See the Nature Portfolio guidelines for submitting code & software for further information.

Data

Policy information about availability of data

All manuscripts must include a data availability statement. This statement should provide the following information, where applicable:

- Accession codes, unique identifiers, or web links for publicly available datasets
- A description of any restrictions on data availability
- For clinical datasets or third party data, please ensure that the statement adheres to our policy

ColVLP sequences were retrieved from the NCBI RefSeq database (downloaded on 02/01/2021; https://www.ncbi.nlm.nih.gov/refseq/) and the PLSDB database (downloaded 23/06/2021; https://ccb-microbe.cs.uni-saarland.de/plsdb2025).
Additional ColVLP co-integrates were retrieved from an NCBI query (performed on 04/04/2024).
Assemblies for 100 STs of E. coli, a E. coli ST95 database (consisting of 2,118 strains; downloaded on 24/05/2021), and an E. coli ST131 database (consisting of 3,857 strains; downloaded on 17/07/2018) were retrieved from Enterobase (https://enterobase.warwick.ac.uk).

Data Availability
The LC-MS data generated in this study has been deposited in the ProteomeXchange Consortium via the PRIDE (96) partner repository under the accession code PXD060817 [http://proteomecentral.proteomexchange.org/cgi/GetDataset?ID=PXD060817]. The metadata for the 233 ColVLP, 100ST, 1377 NCBI RefSeq complete genomes, ST95 draft genomes, and ST131 Clade B draft genomes databases generated in this study are provided in the Source Data file. Sequence data used to verify plasmid constructs is available from the authors upon request. All other data generated in this work is presented in the manuscript.

Research involving human participants, their data, or biological material

Policy information about studies with human participants or human data. See also policy information about sex, gender (identity/presentation), and sexual orientation and race, ethnicity and racism.

Reporting on sex and gender	No human research participants were involved in this study.
Reporting on race, ethnicity, or other socially relevant groupings	No human research participants were involved in this study.
Population characteristics	No human research participants were involved in this study.
Recruitment	No human research participants were involved in this study.
Ethics oversight	No human research participants were involved in this study.

Note that full information on the approval of the study protocol must also be provided in the manuscript.

Field-specific reporting

Please select the one below that is the best fit for your research. If you are not sure, read the appropriate sections before making your selection.

[x] Life sciences [ ] Behavioural & social sciences [ ] Ecological, evolutionary & environmental sciences

For a reference copy of the document with all sections, see nature.com/documents/nr-reporting-summary-flat.pdf

Life sciences study design

All studies must disclose on these points even when the disclosure is negative.

Sample size	Sample sizes were not predetermined based on statistical methods, but were chosen according to the standards in the field - at least 3 independent biological replicates for each condition. This generated sufficient data for statistical analysis.
Data exclusions	ColVLPs which were incomplete or contained ambiguous nucleotides were removed from analyses, as our work only utilised completely sequenced and assembled plasmids.
Replication	Reported results were consistently replicated across multiple experiments (at least three independent biological replicates) with all replicates generating similar results.
Randomization	No randomization was necessary as experiments were performed with appropriate controls. Randomization is not generally used in this field.

Blinding	Investigators were not blinded. Blinding during analysis was not necessary because the results are quantitative and did not require subjective judgment or interpretation. Blinding is not typically used in the field.

Reporting for specific materials, systems and methods

We require information from authors about some types of materials, experimental systems and methods used in many studies. Here, indicate whether each material, system or method listed is relevant to your study. If you are not sure if a list item applies to your research, read the appropriate section before selecting a response.

Materials & experimental systems

n/a	Involved in the study
☒	☐ Antibodies
☒	☐ Eukaryotic cell lines
☒	☐ Palaeontology and archaeology
☒	☐ Animals and other organisms
☒	☐ Clinical data
☒	☐ Dual use research of concern
☒	☐ Plants

Methods

n/a	Involved in the study
☒	☐ ChIP-seq
☒	☐ Flow cytometry
☒	☐ MRI-based neuroimaging

Plants

Seed stocks	No seeds/plants were used in this study
Novel plant genotypes	No seeds/plants were used in this study
Authentication	No seeds/plants were used in this study
